# Evaluation of real-world application of cardiac implantable electronic device-based multi-sensor algorithm for heart failure management

**DOI:** 10.1093/ehjdh/ztaf010

**Published:** 2025-03-11

**Authors:** Jennifer Llewellyn, Rachel Goode, Matthew Kahn, Sergio Valsecchi, Archana Rao

**Affiliations:** Department of Cardiology, Liverpool Heart and Chest Hospital, Liverpool L14 3PE, UK; Department of Cardiology, Liverpool Heart and Chest Hospital, Liverpool L14 3PE, UK; Department of Cardiology, Liverpool Heart and Chest Hospital, Liverpool L14 3PE, UK; Boston Scientific, Viale Forlanini, 23, Milan 20134, Italy; Department of Cardiology, Liverpool Heart and Chest Hospital, Liverpool L14 3PE, UK

**Keywords:** Heart failure, CRT, Remote monitoring, Risk stratification

## Abstract

**Aims:**

Remote monitoring of cardiac implantable electronic devices enables pre-emptive management of heart failure (HF) without additional patient engagement. The HeartLogic™ algorithm in implantable cardioverter defibrillators (ICDs) combines physiological parameters to predict HF events, facilitating earlier interventions. This study evaluated its diagnostic performance and resource implications within an HF management service.

**Methods and results:**

In a single-centre study, 212 patients with cardiac resynchronization therapy ICDs (CRT-Ds) were monitored for 12-months. During follow-up, 18 (8%) patients died, and 15 HF hospitalizations occurred in 13 (6%) patients. Outpatient visits totalled 37 in 34 (16%) patients. HeartLogic™ alerts occurred in 58% of patients, with 100% sensitivity for HF-related hospitalizations. The positive predictive value was 29% including only alerts associated with HF events, while it was 51% including HF events and explained alerts. Unexplained alert rate was 0.46 per patient-year. Clinical interventions, mainly medication adjustments, followed 82 alerts. Total management time was 257 h/year, equivalent to 0.57 full-time equivalents for managing 1000 CRT-D patients.

**Conclusion:**

The integration of HeartLogic™ into routine care demonstrated its utility in optimizing HF management, improving healthcare resource allocation. The algorithm can enhance proactive patient management and provide holistic care within the existing healthcare infrastructure.

## Graphical Abstract

**Graphical Abstract:**
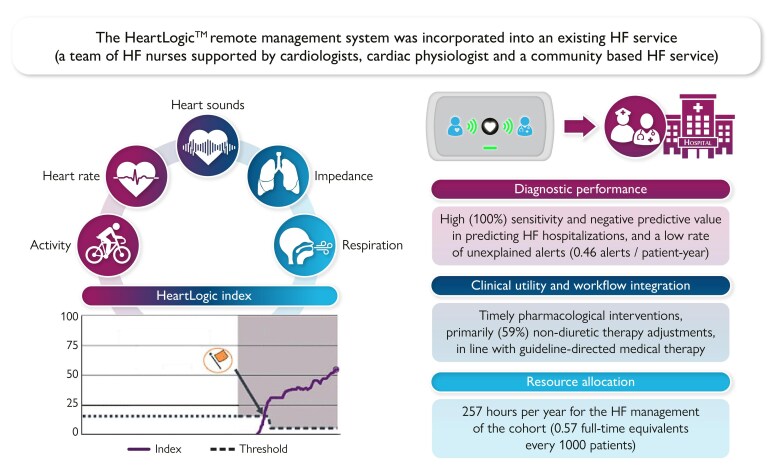

## Introduction

Heart failure (HF) is a complex clinical syndrome associated with significant morbidity and mortality.^1^ Periods of decompensation drive health care utilization^2^ and diminish quality of life.^3^ In the UK, HF is estimated to account for 1 million inpatient bed days annually, representing 5% of all emergency medical admissions and costing ∼£2 billion, or 2% of the National Health Service budget. The majority of these cases are linked to HF with reduced ejection fraction, a condition frequently associated with considerable comorbidity.^4^

The coronavirus pandemic has emphasized the importance of ‘remote’ working, with the vast majority of HF patients in the vulnerable category and shielding. This has led to hesitancy among patients to attend in-person outpatient appointments.^5^ There is significant interest in the use of recorded data from cardiac implantable electronic devices (CIEDs) for predicting (and thus pre-emptively managing) episodes of decompensated HF. An advantage of telemonitoring of CIEDs is that it requires no additional engagement from the patient, as data are gathered automatically and transferred to a cloud-based server. The Implant-based Multiparameter Telemonitoring of Patients with Heart Failure (IN-TIME) randomized clinical trial^6^ demonstrated that daily automatic remote monitoring (RM) enabled early action to be taken in response to the warning signs of acute decompensated HF, resulting in lower all-cause mortality and hospital admission rates for HF. A systematic review of 11 randomized controlled trials showed that RM of CIEDs reduced planned hospital visits and lowered monetary costs, while it did not affect mortality.^2^

The Boston Scientific (Marlborough, MA, USA) high-voltage devices, i.e. implantable cardioverter defibrillators (ICDs) and cardiac resynchronization therapy ICDs (CRT-Ds), are equipped with a multi-parameter HF algorithm to enable better management of the clinical condition. The MultiSENSE trial investigated this novel algorithm named HeartLogic™.^7^ The trial was an observational study in which the physicians were blinded to the algorithm data, therefore no evidence was provided about the impact of its use on HF hospitalization rate. More recently, a study by Treskes *et al*.^8^ suggested that activation of the HeartLogic™ algorithm enables RM of HF patients, resulting in a significant reduction in hospitalizations for decompensated HF, and in health economic benefits.

In the present study, we sought to evaluate the application of the HeartLogic™ algorithm into an existing HF management workflow and understand the resource implications of this.

## Methods

### Study centre

The study was conducted in a cardiac tertiary centre in the Northwest of UK, responsible for the implant and follow up of complex CIEDs for a population of ∼2 million. The follow-up for these devices is performed via RM by cardiac physiologists based at the tertiary centre supported by the cardiologists and specialist HF nurses. All patients with Boston Scientific devices were offered RM of their CRT-D via LATITUDE online patient management platform as per standard of care.

### Patient selection

Consecutive patients who had received a CRT-D (RESONATE family, Boston Scientific) enabled with the HeartLogic™ diagnostic algorithm were eligible for inclusion, provided the device had been implanted for at least 12 months. Patients were excluded if they were <18 years old or pregnant. HeartLogic™ was activated. The study was conducted in accordance with the Declaration of Helsinki, applicable local law, and the European directive for data protection (General Data Protection Regulation). The local institutional ethical committees approved the study prior to commencement.

### Heart logic algorithm

The details of the HeartLogic™ algorithm have been reported previously.^7^ Briefly, the algorithm combines data from multiple sensors: accelerometer-based first (S1) and third (S3) heart sounds, intrathoracic impedance, respiration rate, the ratio of respiration rate to tidal volume, night heart rate, and patient activity. Each day, the device computes a composite index that changes as per changes in clinical parameters from baseline. An alert of impending decompensation is issued and transmitted via the telemonitoring system to the LATITUDE platform when the index crosses a programmable threshold (nominal value 16). A report, showing the trend in the HeartLogic™ index, as well as the relative contributions of the sensors, is provided to the hospital. Weekly transmissions are performed whilst the alert condition persists. The threshold to exit alert is automatically set to a recovery value (nominal value 6).

### Study design

This was a single centre non-blinded single arm study. Enrolled patients had the HeartLogic™ algorithm activated in July 2021 and were followed up for 12 months. Twelve months retrospective data prior to algorithm activation was collected from electronic patient records (EPR).

**Figure 1:**
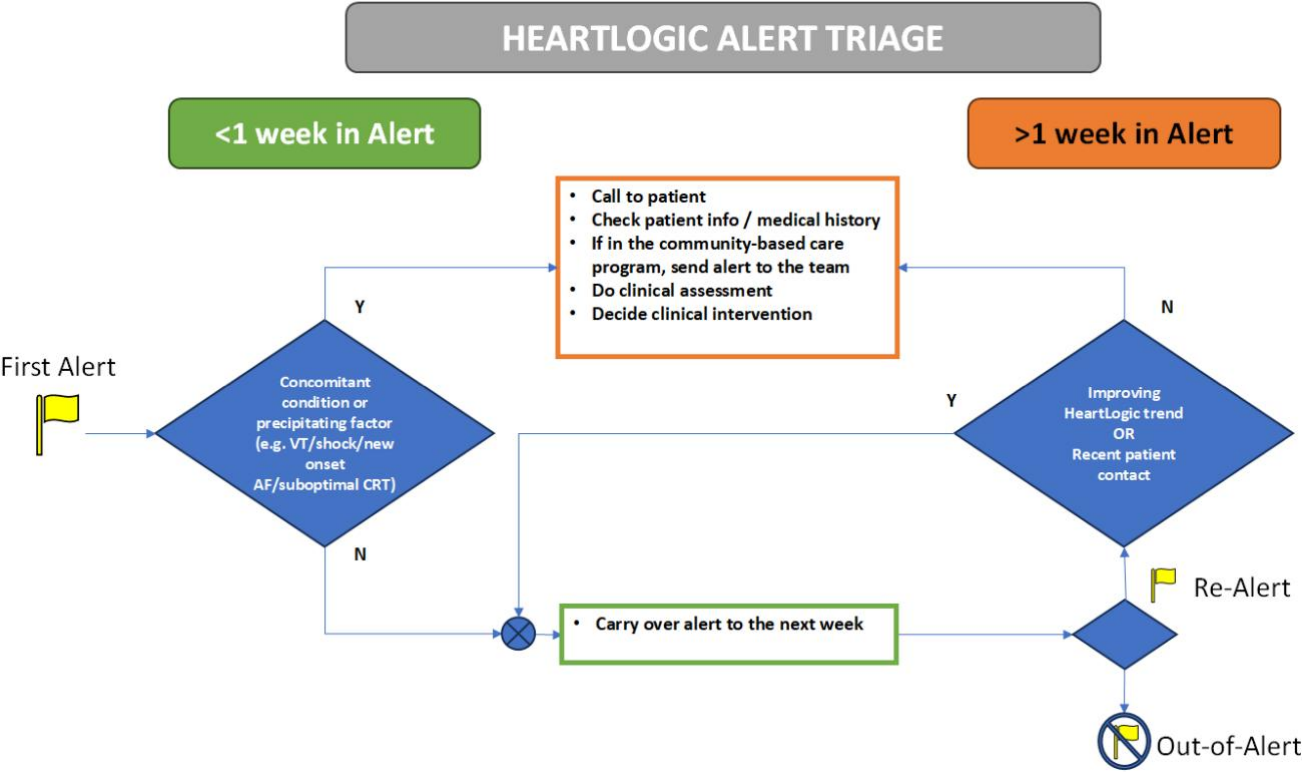
Alert management protocol.

Prior to activation patients were followed up via regularly scheduled remote downloads and outpatient clinic visits, as per the Heart Rhythm Society/European Heart Rhythm Association recommendations.^9^ Following activation, patients were followed up as described with additional review of HeartLogic™ alerts. HeartLogic™ alerts were reviewed weekly by HF specialist nurses with input from the multi-disciplinary team (MDT) as required. The weekly HF MDT included Consultant Cardiologist, Cardiac Physiologists, Clinical Pharmacists, and HF specialist nurse. Each alert was reviewed and actioned as per *Figure 1*. Alerts persisting for <1 week were carried over to the following week. If alerts persisted or revealed clinically significant events such as arrhythmias, patients were contacted by telephone. Standard questions were employed to screen for clinical symptoms. After evaluation, appropriate clinical interventions were initiated, and outcomes were prospectively documented.

Data were collected from actioned alerts, clinical reviews, the LATITUDE database, and EPRs, both prospectively and retrospectively to ensure dataset completeness.

### Resource implications

A 2-months run-in period was employed to streamline processes. Once the pathway was established, the time allocated to each activity related to HeartLogic™-triggered transmission management was documented and the estimate was drawn for a 12-month period. The total time spent was determined by multiplying the average time spent on reviewing and actioning alerts by the number of transmissions, and then adding the time spent on general activities. The estimate was derived from the population actually managed at the centre but was then recalculated to reflect a standard sample of 1000 patients with CRT-D.

### Statistical analysis

Sensitivity was defined as the ratio between the number of HF hospitalizations detected and the total number of hospitalizations. The positive predictive value was defined as the proportion of alerts that were positively associated with HF events. Negative predictive value was defined as the proportion of individual days associated with HF events among those in which HeartLogic was not in alert state.^7^ Alerts were defined as unexplained if not associated with clinical events/symptoms and false-positive if not associated with HF symptoms and were reported in terms of rates per patient-year. In our analysis, we considered signs or symptoms of HF and the corrective therapies delivered (e.g. oral medication changes) to prevent HF events. We assessed the events that occurred in the 12-month period after the HeartLogic™ activation.

Quantitative variables are reported as means ± SD if normally distributed, or medians with 25th–75th percentiles in the case of skewed distribution. Normality of distribution was tested by means of the non-parametric Kolmogorov–Smirnov test. Categorical data are expressed as percentages. Differences between mean data were compared by means of a *t*-test for Gaussian variables, using the F test to check the hypothesis of equality of variance. The Mann–Whitney non-parametric test was used to compare non-Gaussian variables. Differences in proportions were compared by applying χ^2^ analysis or Fisher’s exact test, as appropriate. One-way analysis of variance was used to test for differences among groups. Clinical event rates were expressed as events per patient-year and were calculated as the ratio between the total count of events and the respective duration of the period. The rates were calculated for the entire observation period, i.e. the sum of all patients’ follow-up period, and for the sum of all in-alert period. The in-alert period started when the HeartLogic index crossed the threshold and ended when the index decreased to below the recovery threshold (or at the end of follow-up).^10^ A *P*-value <0.05 was considered significant for all tests. All statistical analyses were performed by means of R: a language and environment for statistical computing (R Foundation for Statistical Computing, Vienna, Austria).

## Results

### Study population and follow-up

In July 2021, HeartLogic™ was activated in 212 consecutive patients who received a CRT-D at least 12 months prior. The median time between implantation and algorithm activation was 28 months (25th–75th percentile: 21–34). The HeartLogic™ index threshold was programmed to the nominal value of 16 in all patients and was not modified during follow-up. *Table 1* shows the baseline clinical variables of all patients in the present analysis.

**Table 1:** Demographics and baseline clinical parameters of the study population and of the groups with and without clinical events and HeartLogic™ alerts during the observation period

Parameter	Total (212)	Death or HF hospitalization (26)	HeartLogic™ alert (124)
Male gender, *n* (%)	169 (80)	23 (88)	101 (81)
Age, years	70 ± 10	74 ± 8*	71 ± 10
Ischaemic aetiology, *n* (%)	91 (43)	15 (58)	63 (51)
NYHA class			
Class I, *n* (%)	13 (6)	0 (0)	3 (2)
Class II, *n* (%)	134 (63)	14 (54)	72 (58)
Class III, *n* (%)	65 (31)	12 (46)	49 (40)
Clinical Frailty Scale	3.3 ± 1.1	4.4 ± 1.3*	3.5 ± 1.2**
HF hospitalizations in the 12 months before activation, *n* (%)	10 (5)	2 (8)	7 (6)
LV ejection fraction ≤35%, *n* (%)	185 (87%)	24 (92)	107 (86)
QRS duration, ms	154 ± 20	154 ± 21	156 ± 19**
AF history, *n* (%)	102 (48)	17 (65)	72 (82)**
Creatinine level (mg/dL)	1.08 ± 0.41	1.28 ± 0.60	1.13 ± 0.43**
eGFR, mL/min	62 ± 18	54 ± 20	59 ± 18**
Diabetes, *n* (%)	62 (29)	11 (42)	38 (31) **
COPD, *n* (%)	33 (16)	10 (38)*	21 (17)
β-Blocker use, *n* (%)	195 (92)	22 (85)	116 (94)
ACE-I or ARB use, *n* (%)	135 (64)	17 (65)	81 (65)
ARNI use, *n* (%)	67 (32)	4 (15)	34 (27)
MRA use, *n* (%)	150 (71)	17 (65)	88 (71)
Diuretic use, *n* (%)	136 (64)	21 (81)	89 (72)**
SGLT2 inhibitors use, *n* (%)	21 (10)	2 (8)	8 (6)**
Secondary prevention, *n* (%)	37 (17)	3 (12)	24 (19)
Biventricular pacing percentage ≥90%, (%)	183 (86)	21 (81)	107 (86)

NYHA, New York Heat Association; HF, heart failure; LV, left ventricular; AF, atrial fibrillation; eGFR, estimated glomerular filtration rate; COPD, chronic obstructive pulmonary disease; ACE-I, angiotensin-converting-enzyme inhibitors; ARB, angiotensin II receptor blockers; ARNI, angiotensin receptor-neprilysin inhibitor; MRA, mineralocorticoid receptor antagonist; SGLT2, sodium-glucose cotransporter-2.

^*^ *P* < 0.05 vs. no death or HF hospitalizations.

^**^ *P* < 0.05 vs. no HeartLogic™ alert.

### Clinical outcomes

During the 12 months post activation, 18 (8%) patients died, and 47 hospitalizations were reported in 43 (20%) patients. HF was the primary diagnosis for 15 (32%) hospitalizations in 13 patients, with a median length of stay of 5 days (25th–75th percentile: 1–7).

The total number of outpatient visits was 37 in 34 (16%) patients. *Table 1* shows the baseline clinical variables of patients with death or HF hospitalizations during the observation period.

### HeartLogic™ alerts

**Figure 2:**
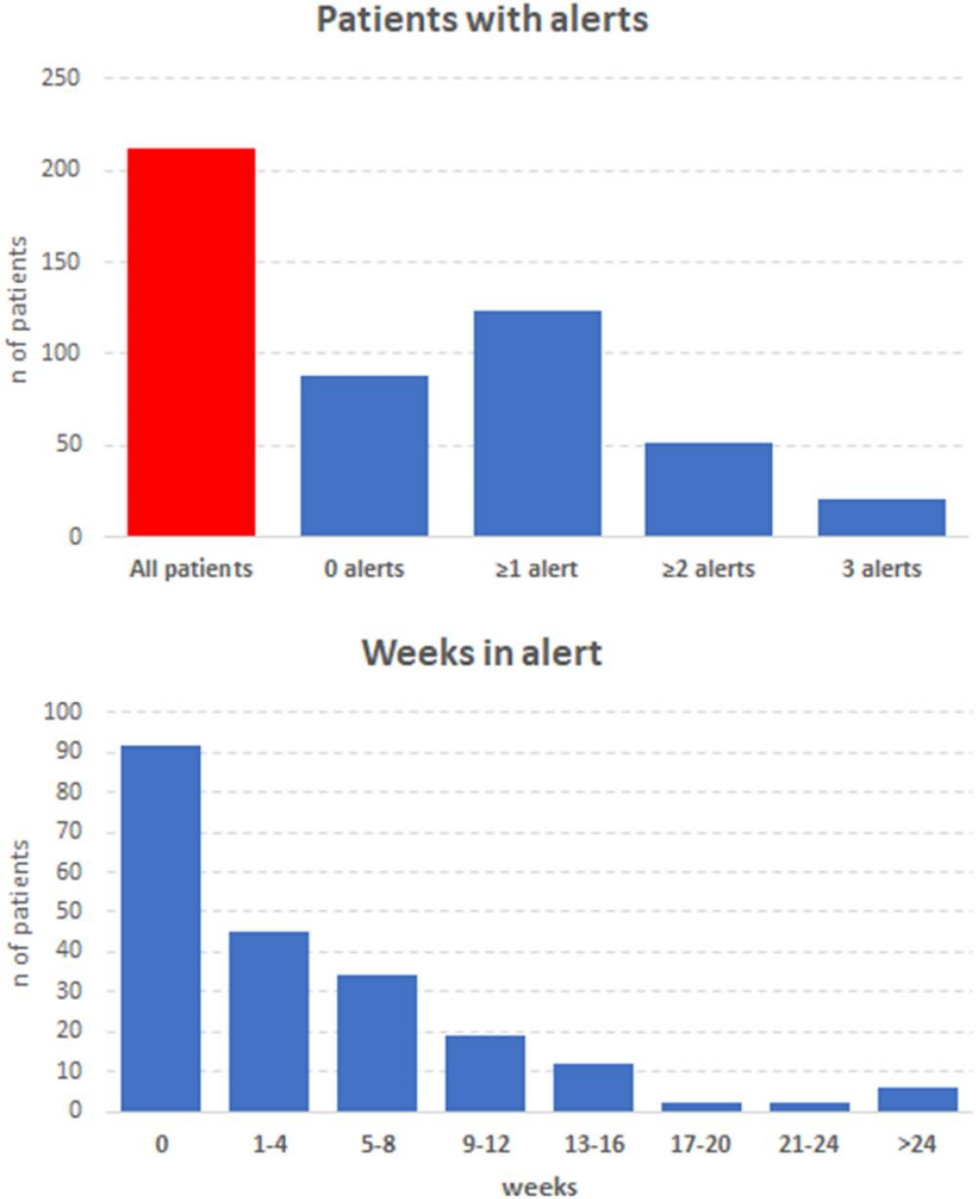
Distribution of patients according to the number of alerts and the time in alert.

During the first 12 months after activation, the HeartLogic™ index crossed the threshold value 197 times in 124 (58%) patients. The alert rate was 0.95 alerts/patient-year in the overall population and 1.63 alerts/patient-year in the group of patients with ≥1 alert. *Figure 2* shows the distribution of patients according to the number of alerts. *Table 1* shows the baseline clinical variables of patients with alerts during the observation period. The average duration of HeartLogic™ alert periods (from index threshold crossing to decrease below the recovery threshold) was 36 ± 29 days. *Figure 2* also shows the distribution of patients according to the time in alert.

Overall, the time in the alert state was 9% of the total observation period (12 months or time to death). All HF hospitalizations occurred during the HeartLogic™ alert state period, resulting in 100% negative predictive value. No HF hospitalization occurred in the absence of a HeartLogic alert, resulting in a 100% sensitivity. The rate of HF hospitalizations in the HeartLogic™ alert state was 0.82 events/patient-year. Overall, 101 (51%) alerts were associated with clinical signs or symptoms, while the remaining 96 (49%) alerts were unexplained (0.46 alerts/patient-year). The number of alerts not associated with HF signs or symptoms, or corrective therapies was 125 (63%), resulting in a false positive rate of 0.60 alerts/patient-year. The positive predictive value was 29% (57/197) including only alerts associated with HF events, while it was 51% (101/197) including both alerts associated with HF events and explained alerts.

### Clinical management

In response to 82 alerts, specific therapeutic actions were implemented, including changes in medical therapy in 61 (74%) cases, 25 of which involved increases in diuretics. Other common interventions included 7 (9%) cases of radiofrequency ablation for atrial fibrillation and 4 (5%) sessions of device reprogramming. Out of the 197 HeartLogic™ alerts, 31 (16%) necessitated hospital admission for clinical condition management. Management of 104 alerts (53%) involved outpatient visits or telephone contacts following remote data review.

**Figure 3:**
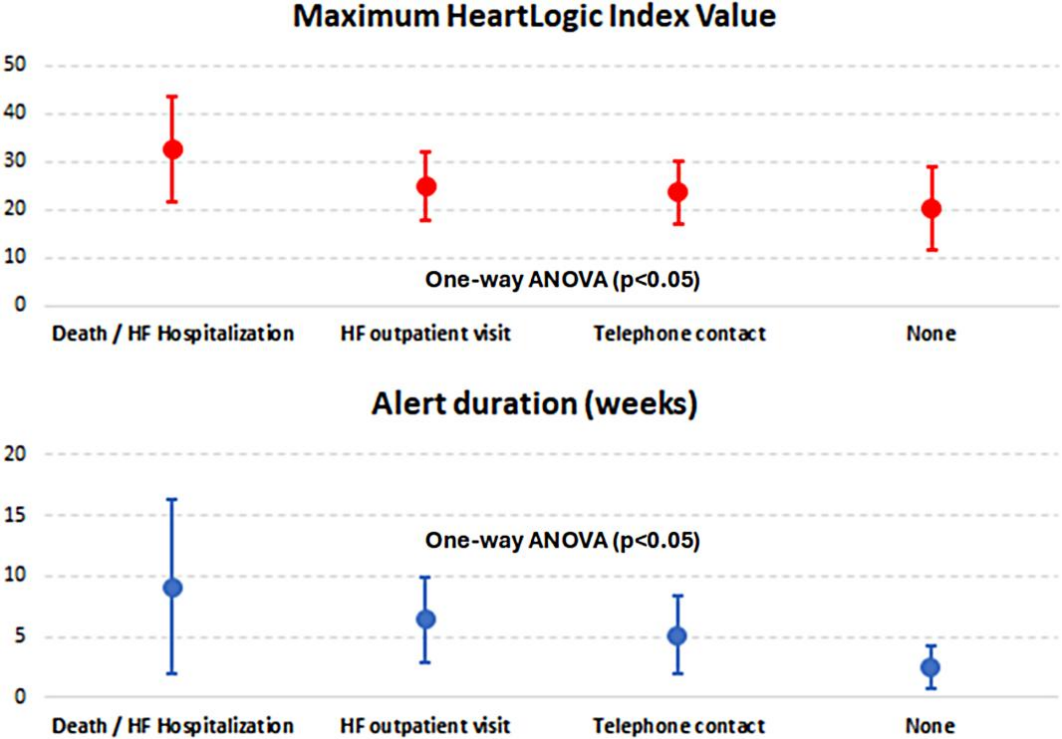
Maximum HeartLogic™ index value and duration of the alert periods stratified for the type of intervention required.

The remaining alerts did not prompt patient contact due to their short duration (27 alerts lasting <1 week), because they occurred within 4 weeks of the last contact, or because of no response from the patient (*Table 2*). The maximum HeartLogic™ index value and the duration of the alert periods stratified for the type of intervention required is reported in *Figure 3*.

**Table 2:** Frequency of signs and symptoms at the time of alerts, and actions taken to manage the condition detected

	Actions reported	No actions reported
HF signs or symptoms	51 (26)	6 (3)
non-HF-related signs or symptoms	16 (8)	13 (7)
Asymptomatic	15 (8)	96 (49)
**Type of admission/contact**		
Hospital admission	31 (16)	—
Outpatient visit	41 (21)	—
Telephone call	63 (32)	—
Telephone call attempt—No answer	19 (10)	—
No call	43 (22)	—

### Time allocation

**Figure 4:**
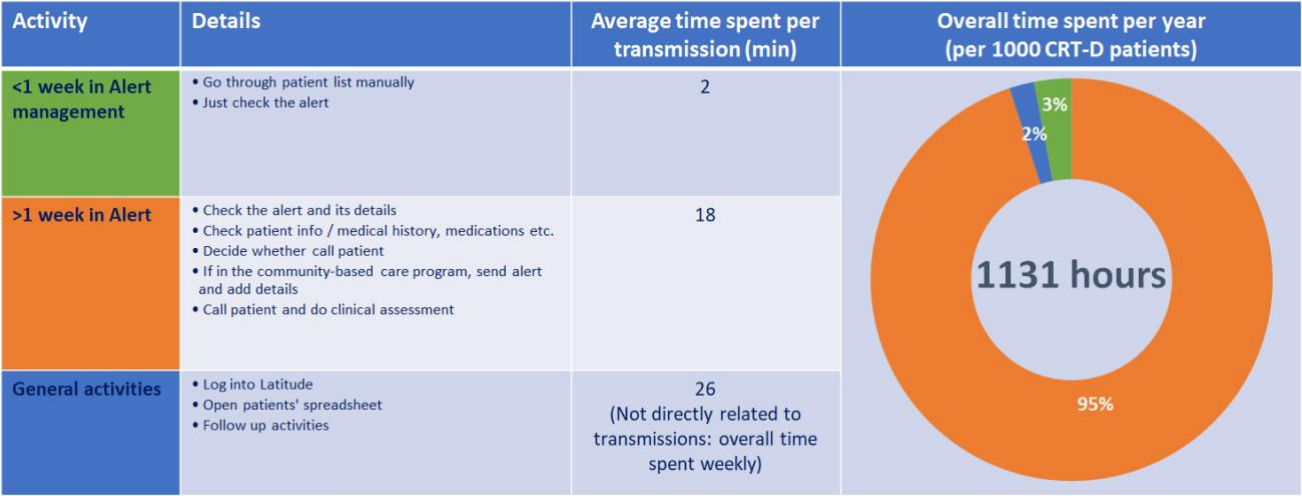
HeartLogic™-triggered transmission management activities and average time spent.

The overall number of HeartLogic™-triggered transmissions (first alerts and weekly re-alerts) was 957 during the observation period. The transmission management activities and the time allocated are reported in *Figure 4*. The overall time spent for the management of the cohort was 257 h/year. This translates into ∼1131 h (i.e. 0.57 full-time equivalents) for the HF management of 1000 patients with CRT-D via the adopted HeartLogic™ protocol.

## Discussion

This manuscript presents the real-world experience of CIED patients with HeartLogic™ algorithm incorporated into an existing HF management workflow.

Our experience confirmed the diagnostic performance of the algorithm demonstrated in the validation study.^7^ Specifically, in our patients with CRT-D devices, we reported a rate of ∼1 HeartLogic™ alert per patient-year, consistent with previous studies.^11,12^ In the MultiSENSE trial,^7^ operators were blinded to the diagnostics and could not take any clinical action in response to alerts, resulting in patients spending 17% of their time in alert. In our study, the time spent in alert was lower at 9%, aligning with the 11% value reported in the Re-HEART study.^13^ We believe that this is a consequence of our management protocol aimed at intervening promptly to reduce HF decompensation duration and time spent in alert.

In our experience, the algorithm’s sensitivity was 100%, anticipating all HF hospitalizations by algorithm alerts. This confirms previous findings^11,13^ and early clinical practice observations.^14^ Combined with the algorithm’s ability to stratify HF event risk,^10,12^ this suggests its utility in discriminating patients during higher-risk periods, optimizing healthcare resource utilization. With the growing burden on healthcare resources, there is a trend towards alert-based follow-up in RM of CIEDs.^9^ The strong negative predictive value of HeartLogic™ previously demonstrated^7,13^ and reaffirmed here, extends its applicability to HF management, and could increase alert-based HF CIED management adoption.

Our study documented a rate of 0.46 unexplained alerts per patient-year highlighting a complex and comorbid cohort. The prospective MultiSENSE study reported an unexplained alert rate of 1.47 alerts per patient-year,^7^ while the RE-HEART Registry yielded results consistent with ours in terms of unexplained alerts.^13^ As previously reported,^14,15^ alerts were more frequent in patients with atrial fibrillation, worse renal function, and diabetes, yet these variables were not associated with clinical events, emphasizing alerts’ independent predictive value. The positive predictive value was relatively low at 51%, influenced in part by the low incidence of HF events. This aligns with previous findings from the MultiSENSE study (11.3%) and the RE-HEART Registry (57%).^7,13^ The algorithm is designed to support healthcare professionals in reviewing HF data rather than serving as a standalone tool for treatment decisions. Consequently, unnecessary treatments resulting from false-positive alerts are unlikely, and the risk of harm due to overtreatment is expected to be minimal.^16^

We detailed how often alerts correlated with signs/symptoms or implemented clinical actions during patient contacts per protocol. As per our alert management protocol, we recommended intervention only for alerts persisting over 1 week or with non-recent patient contacts, this has been previously described by Treskes *et al*.,^8^ de Juan Bagudá *et al*.,^13^ and Feijen *et al*.^17^ with a view to improve workflow efficiency. We noted that 21% of the alerts were in their first week and as a result only contributed to 3% of the workload. The additional workload should ideally be offset by a potential reduction in the need for unplanned HF-related hospital visits and, hopefully, shorter hospitalizations, as previously suggested.^8,17^

At our centre, where patients are carefully monitored by a team of cardiac physiologists supported by the cardiologists, specialist nurses, and are frequently included in a community-based care program, therapeutic interventions triggered by alerts principally involved pharmacological adjustments. In our cohort, 59% were non-diuretic pharmaceutical adjustments, primarily optimization of guideline-directed medical therapy for HF. This is consistent with the treatment algorithm outlined in the MANAGE-HF study.^18^ This has further been endorsed by Boriani *et al*.^19^ recommending escalating disease-modifying agents in response to impending HF alerts from devices in the absence of clinical signs of congestion.

The impact of implementing the HeartLogic™ remote management into an existing HF service was assessed. A wide range of interventions was delivered as a result of alerts. This included opportunistic optimization of guideline directed medical therapy, delivery of LV pacing, rhythm management, prognostication, and end of life care.

There is emerging evidence for improved patient outcomes when utilizing HeartLogic™-guided management protocol.^8,12,17^ Interestingly, our data demonstrate the correlation between poorer outcomes and the maximum HeartLogic index value, along with alert duration. This appears to validate the potential prognostic value of the algorithm.^20^

Our study describes a safe clinical pathway for incorporation of HeartLogic into an existing HF CIED service and provides a practical insight into resource allocation within the NHS for a sustainable service.

### Limitations

The main limitation of this study is its observational, non-controlled, and non-randomized design.

## Conclusions

The strong negative predictive value of HeartLogic™ allows for confidence in alert-based follow-up of HF patients with CIEDs *in situ* for not just the management of their devices but also for HF condition.

HeartLogic alert-based follow-up offers a unique opportunity for proactive patient management and holistic HF care.

## Data Availability

The experimental data used to support the findings of this study are available from the corresponding author upon reasonable request.

## References

[1] McDonagh TA, Metra M, Adamo M, Gardner RS, Baumbach A, Böhm M, et al 2021 ESC guidelines for the diagnosis and treatment of acute and chronic heart failure. Eur Heart J 2021;42:3599–3726. 34447992 10.1093/eurheartj/ehab368

[2] Klersy C, Boriani G, De Silvestri A, Mairesse GH, Braunschweig F, Scotti V, et al Effect of telemonitoring of cardiac implantable electronic devices on healthcare utilization: a meta-analysis of randomized controlled trials in patients with heart failure. Eur J Heart Fail 2016;18:195–204. 26817628 10.1002/ejhf.470

[3] Nieminen MS, Dickstein K, Fonseca C, Serrano JM, Parissis J, Fedele F, et al The patient perspective: quality of life in advanced heart failure with frequent hospitalisations. Int J Cardiol 2015;191:256–264. 25981363 10.1016/j.ijcard.2015.04.235

[4] National Heart Failure Audit: 2024 Summary Report (2022/23 Data). London, UK: National Institute for Cardiovascular Outcomes Research (NICOR); 2024.

[5] Sankaranarayanan R, Hartshorne-Evans N, Redmond-Lyon S, Wilson J, Essa H, Gray A, et al The impact of COVID-19 on the management of heart failure: a United Kingdom patient questionnaire study. ESC Heart Fail 2021;8:1324–1332. 33463044 10.1002/ehf2.13209 PMC8006619

[6] Hindricks G, Taborsky M, Glikson M, Heinrich U, Schumacher B, Katz A, et al Implant-based multiparameter telemonitoring of patients with heart failure (IN-TIME): a randomised controlled trial. Lancet 2014;384:583–590. 25131977 10.1016/S0140-6736(14)61176-4

[7] Boehmer JP, Hariharan R, Devecchi FG, Smith AL, Molon G, Capucci A, et al A multisensor algorithm predicts heart failure events in patients with implanted devices: results from the MultiSENSE study. JACC Heart Fail 2017;5:216–225. 28254128 10.1016/j.jchf.2016.12.011

[8] Treskes RW, Beles M, Caputo ML, Cordon A, Biundo E, Maes E, et al Clinical and economic impact of HeartLogic™ compared with standard care in heart failure patients. ESC Heart Fail 2021;8:1541–1551. 33619901 10.1002/ehf2.13252 PMC8006675

[9] Ferrick AM, Raj SR, Deneke T, Kojodjojo P, Lopez-Cabanillas N, Abe H, et al 2023 HRS/EHRA/APHRS/LAHRS expert consensus statement on practical management of the remote device clinic. Europace 2023;25:euad123. 37208301 10.1093/europace/euad123 PMC10199172

[10] Gardner RS, Singh JP, Stancak B, Nair DG, Cao M, Schulze C, et al HeartLogic multisensor algorithm identifies patients during periods of significantly increased risk of heart failure events: results from the MultiSENSE Study. Circ Heart Fail 2018;11:e004669. 30002113 10.1161/CIRCHEARTFAILURE.117.004669

[11] Santini L, D'Onofrio A, Dello Russo A, Calò L, Pecora D, Favale S, et al Prospective evaluation of the multisensor HeartLogic algorithm for heart failure monitoring. Clin Cardiol 2020;43:691–697. 32304098 10.1002/clc.23366 PMC7368302

[12] Calò L, Bianchi V, Ferraioli D, Santini L, Dello Russo A, Carriere C, et al Multiparametric implantable cardioverter-defibrillator algorithm for heart failure risk stratification and management: an analysis in clinical practice. Circ Heart Fail 2021;14:e008134. 34190592 10.1161/CIRCHEARTFAILURE.120.008134 PMC8522625

[13] de Juan Bagudá J, Gavira Gómez JJ, Pachón Iglesias M, Cózar León R, Escolar Pérez V, González Fernández Ó, et al Remote heart failure management using the HeartLogic algorithm. RE-HEART registry. Rev Esp Cardiol (Engl Ed) 2022;75:709–716. 34896031 10.1016/j.rec.2021.09.015

[14] Capucci A, Santini L, Favale S, Pecora D, Petracci B, Calò L, et al Preliminary experience with the multisensor HeartLogic algorithm for heart failure monitoring: a retrospective case series report. ESC Heart Fail 2019;6:308–318. 30632306 10.1002/ehf2.12394 PMC6437441

[15] Santobuono VE, Favale S, D'Onofrio A, Manzo M, Calò L, Bertini M, et al Performance of a multisensor implantable defibrillator algorithm for heart failure monitoring related to co-morbidities. ESC Heart Fail 2023;10:2469–2478. 37278122 10.1002/ehf2.14416 PMC10375157

[16] National Institute for Health and Care Excellence (NICE) . Heart Failure Algorithms for Remote Monitoring in People with Cardiac Implantable Electronic Devices. Diagnostics Guidance [DG61]. London: NICE; 2024.

[17] Feijen M, Beles M, Tan YZ, Cordon A, Dupont M, Treskes RW, et al Fewer worsening heart failure events with HeartLogic on top of standard care: a propensity-matched cohort analysis. J Card Fail 2023;29:1522–1530. 37220824 10.1016/j.cardfail.2023.04.012

[18] Hernandez AF, Albert NM, Allen LA, Ahmed R, Averina V, Boehmer JP, et al Multiple cArdiac seNsors for mAnaGEment of heart failure (MANAGE-HF)—phase I evaluation of the integration and safety of the HeartLogic multisensor algorithm in patients with heart failure. J Card Fail 2022;28:1245–1254. 35460884 10.1016/j.cardfail.2022.03.349

[19] Boriani G, Imberti JF, Bonini N, Carriere C, Mei DA, Zecchin M, et al Remote multiparametric monitoring and management of heart failure patients through cardiac implantable electronic devices. Eur J Intern Med 2023;115:1–9. 37076404 10.1016/j.ejim.2023.04.011

[20] D'Onofrio A, Vitulano G, Calò L, Bertini M, Santini L, Savarese G, et al Predicting all-cause mortality by means of a multisensor implantable defibrillator algorithm for heart failure monitoring. Heart Rhythm 2023;20:992–997. 36966948 10.1016/j.hrthm.2023.03.026

